# Supplementary material for: Venomix: a simple bioinformatic pipeline for identifying and characterizing toxin gene candidates from transcriptomic data
Source: PeerJ. 2018 Jul 31;6:e5361. doi: 10.7717/peerj.5361 (PMC6074769; doi:10.7717/peerj.5361)
Supplement: Supplemental Information 6 [file peerj-06-5361-s006.gz › FinalOutput_1E-6/Toxin_Pg8_10/finaltree.pdf]

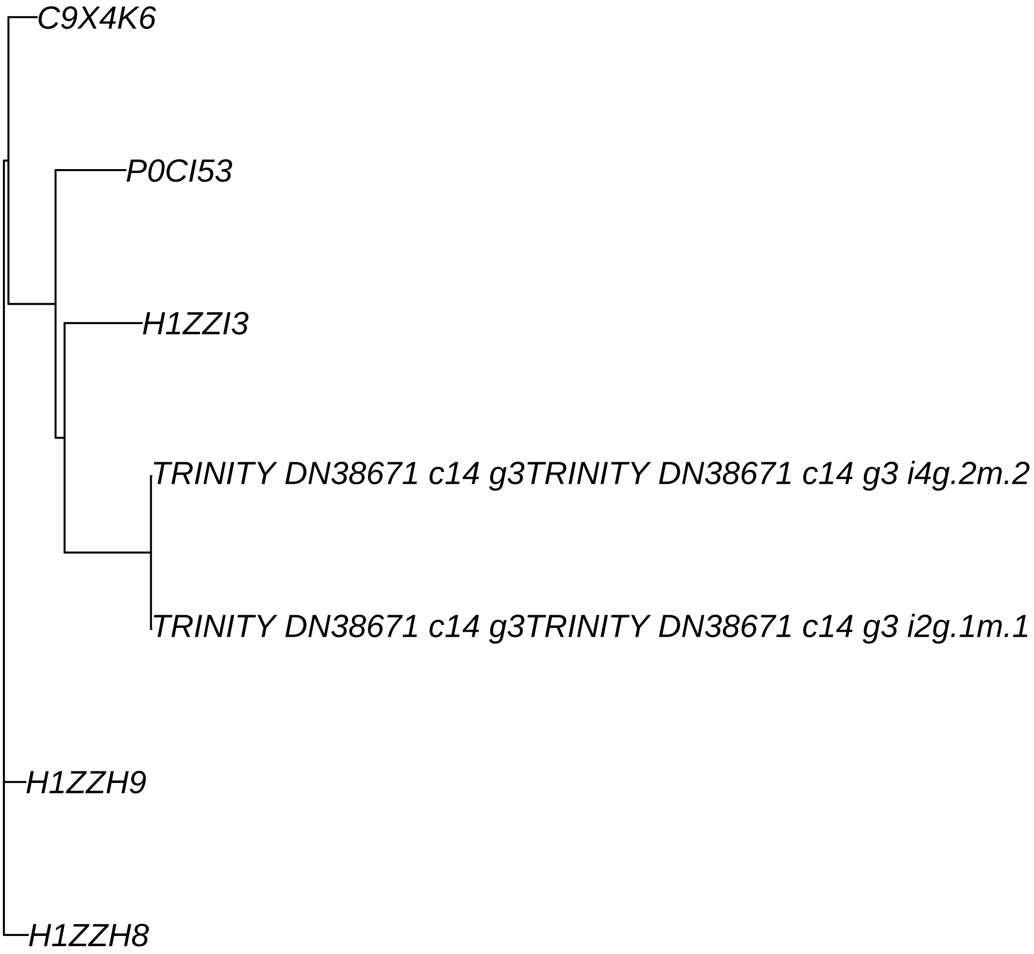
C9X4K6
P0CI53
H1ZZI3
TRINITY DN38671 c14 g3TRINITY DN38671 c14 g3 i4g.2m.2
TRINITY DN38671 c14 g3TRINITY DN38671 c14 g3 i2g.1m.1
H1ZZH9
H1ZZH8
